# Global trends and research hotspots on HACCP and modern quality management systems in the food industry

**DOI:** 10.1016/j.heliyon.2023.e18232

**Published:** 2023-07-15

**Authors:** Elena Radu, Adriana Dima, Ecaterina Milica Dobrota, Ana-Maria Badea, Dag Øivind Madsen, Cosmin Dobrin, Silvius Stanciu

**Affiliations:** a Faculty of Business Administration, The Bucharest University of Economic Studies, 010374, Bucharest, Romania; b Faculty of Management, The Bucharest University of Economic Studies, 010374, Bucharest, Romania; c Department of Business, Consumer Sciences and Quality Management, The Bucharest University of Economic Studies, 010374, Bucharest, Romania; d USN School of Business, University of South-Eastern Norway, 3511 Hønefoss, Norway; e Faculty of Food Science and Engineering, “Dunărea de Jos” University of Galați, 800008, Galați, Romania

**Keywords:** Bibliometric, Food industry, Food safety, Food science, HACCP, Quality management control, Quality management system

## Abstract

HACCP (Hazard Analysis and Critical Control Points) and modern quality management systems have a significant impact on public health in the food industry. These systems ensure that food products are safe for consumption by identifying and managing potential hazards at every stage of the production process. To stimulate ongoing studies in both developing and underexplored areas of inquiry, this research synthesizes and organizes the contributions made in this field. It examines more than 40 years of studies from Scopus data base on HACCP and modern quality management systems in the food industry using the VOSviewer software version 1.6.18 (Leiden University, The Netherlands) and bibliometrix R-package. This represents, to the authors’ knowledge, the first bibliometric analysis undergone in this direction. The graphical framework demonstrates the highest developments in research and the literature review investigates barriers and opportunities of implementing HACCP in food industry organizations. Findings indicate that until the beginning of the 1990s, there was not a large number of scientific production in the field of HACCP and modern quality management systems in the food industry. The USA were the most prolific affiliation terms of scientific production until 2012, when studies from Italy, the United Kingdom, China and Greece intensified. Currently, the most prolific country in terms of publications is Italy. In terms of global cooperation, the United Kingdom, The United States and The Netherlands represent most active nations on this topic Motor themes that reflect the main interest of the researchers include food diseases, quality control, hazards or food supply. The study also provides future research directions regarding food quality and safety management. These should be focused on improving the safety, quality, and sustainability of food products, while also adapting to changing consumer demands, emerging risks, and regulatory requirements.

## 1 Introduction

The food industry plays an important part in the economy of the European Union and other countries, as food is a vital element of any nation. Regardless of their position in the food chain, product safety represents a very important concern for all traders. Also, consumers aware of the link between food, diet and health are increasingly interested in safe food [1]. The companies achieve a high degree of food safety by using control instruments for the hazards to which products are exposed [2]. HACCP is considered, worldwide, the best food safety management system [3]. The HACCP system, developed by NASA to ensure the safety of food included in space programs has subsequently been implemented in international standards in all food industry activities concerning the production, transport, packaging, storage or marketing of food products [4]. According to Directive 93/43, all economic operators involved in the food industry are responsible for the analysis of hazards and critical control points (HACCP) in order to be able to supply safe food to the consumer market [5].

Through Regulation no. 852/2004 the general hygiene established requirements that must be respected throughout the food chain [6]. The regulation shows that, from the perspective of food hygiene, food safety is the result of a number of factors: the establishment by means of legislation of minimum hygiene requirements, the implementation of official controls, in order to determine the way of compliance with the legislation in the field by economic operators, the application by companies in the food sector of food safety procedures based on HACCP principles.

In Cyprus, with the assistance of consultancy firms, the government helped food companies implement the Food Hygiene Directive 93/43/EEC following EU accession in 2004 [7]. In Serbia, the government has also supported food businesses to adopt HACCP [1].

By applying HACCP requirements for SMEs that provide services in this field, in South Korea, Lee and Hong [8] determined 15 factors of significant importance, the relevant ones being: “checking and controlling pests and rodents”, “preventing cross-contamination of food”, “employee uniforms cleanliness and hand washing”, “recording results of improved factors”. In the food distribution flow, packaging and transportation are critical control points in HACCP plan analysis, as Twede and Harte, 2011, found in their study in the US [9].

The implementation of the food quality standards is not an easy task for companies, especially if they come under the small business category where the number of employees is small. The application of HACCP analysis, especially the establishment of critical control points and identification of hazards, requires thorough skills and knowledge on the part of quality managers [10]. In this regard, food companies need to nominate qualified, well-trained managers who have a thorough knowledge of the principles of HACCP [11].

The HACCP aims to monitoring the entire production flow in order to achieve a safe product according to the applicable standards [12]. The benefit of implementing this food safety management system is both the achievement of higher quality products and the reduction of disease transmission through food [13]. Altemimi and other entities analyzed the level of microbial contamination of various types of surfaces in contact with food, as well as the effectiveness of disinfectants on contaminated surfaces, concluding that the application of HACCP principles in Iraq improved food quality [14].

Rosak-Szyrocka and Abbase [10], through their study of the ice cream production process in Poland, showed that the finished product must be accompanied by correct records, it is necessary that the traceability of production is established at all times, from the raw material supplier to the customer. By researching the apple juice concentrate process in China, Duan and other entities established a proper HACCP plan so that the level of patulin contamination of the product could be reduced [15].

Following the technical approach to the implementation of the management system at the level of a company in Kazakhstan, Akhmetova, Fuschi and Vasiliunaite [16] noted that compliance with HACCP principles leads the enterprise towards meeting the conditions and requirements of the market ensuring competitive products. Costa and other entities analyzed the activity in abattoirs in Argentina and found that by implementing the HACCP plan, zero tolerance requirements for Salmonella in beef can be met [17].

The “farm to fork” principle requires all companies in the food industry to rethink the way they implement their food quality management system in order to comply with sustainability criteria in the food chain [18].

Lack of knowledge of the HACCP benefits, lack of managers' motivation to implement this system, lack of company staff training are barriers to the adoption of HACCP in the food industry. In addition, there are the high costs associated with the correct implementation of HACCP. The managers of food businesses need to be aware that the application of HACCP rules leads to increased performance of production processes, reduced losses and therefore lower costs. The improvements of customer relationships, as well as an increase in the customer portfolio and the firm's prestige are the benefits of running the company's business according to HACCP rules [13]. Analyzing the impact of HACCP on the Jamaican hotel industry, Fletcher, Maharaj and James [19] concluded that the compliance with food standards is reflected in the appreciation given by tourists, but also that the implementation of the standards requires specific policies tailored to different types of hotels. In Italy, through research in the fish industry, it was determined that the establishment of hazards and critical control points in the fish processing flow (from fishing to inclusion in food) facilitates communication and standardization of processes in this area [20].

The main research questions of the current study are the following:

RQ1 What are the studied barriers of implementing HACCP as modern quality management system in food industry organizations and through what research methodologies?

RQ2 What are the explored opportunities of implementing HACCP as modern quality management system in food industry organizations and through what research methodologies?

RQ3 What is the frequency of scientific production on HACCP and modern quality management systems in the food industry?

RQ4 Which are the most important journals, countries, research organizations in terms of scientific knowledge on HACCP and modern quality management systems in the food industry?

RQ5 What are the main research clusters referring to HACCP and modern quality management systems in the food industry?

RQ6 What are the main future research directions regarding the analyzed topic?

The novelty and most important contribution of this research resides in the fact that it represents the first bibliometric investigation of HACCP and modern quality management systems in the food industry. Furthermore, another major feature of this study is that it offers a thorough evaluation of barriers and opportunities of implementing HACCP as modern quality management system in food industry organizations.

In order to investigate novel approaches in the field of on HACCP and modern quality management systems in food industry, researchers may find this study useful because it offers a general grasp of the completely investigated domain. Additionally, this research can help organizations in the food industry, public and private universities, as well as research centers, gain a deeper understanding of the future paths for the development of HACCP and other modern quality management systems in the food domain.

## 2 Literature review

The development and implementation of management systems that would guarantee the safety of food products for the consumer and also provide the required and stable quality of products is becoming increasingly important in the food industry of all countries as a result of the growing perception that food products pose a significant health risk. The challenge of adjusting to the realities of a market economy is one of the most pressing issues facing businesses today. When it comes to today's business climate, a company's ability to stay afloat and maintain a strong position in the goods market is directly proportional to its degree of competitiveness. Consequently, competitiveness is linked to price stability, product quality, worker productivity, and resource conservation. Doing so is feasible, provided the business in question employs a tried-and-true quality management system [21].

### 2.1 The quality management system

Food can be affected by a number of pathogenic factors that will alter its physical, chemical and sensory quality. Whether they originate from the overuse of pesticides, the environment, the water, the production process, the transport or handling processes, these pathogens are detrimental to health. The food safety can be ensured through food quality control.

At the EU level, the Directive 93/43/EEC obliges economic operators, using the HACCP method, to identify essential food safety activities, to establish, to maintain and to review the necessary safety procedures. All this is part of the quality management system (QMS) and its formulation is the responsibility of each food company [22]. This system is intended, on the one hand, for the organizations that implement it for their own documentation of internal processes, but also for the description of activities (with the establishment of critical points and control areas, for the characterization of measurable elements, the development of working procedures, etc.). On the other hand, this QMS is intended for partner companies, external to the organization, the latter ensuring that in conducting its business it collaborates with companies for which food quality is paramount.

**Fig. 1:**
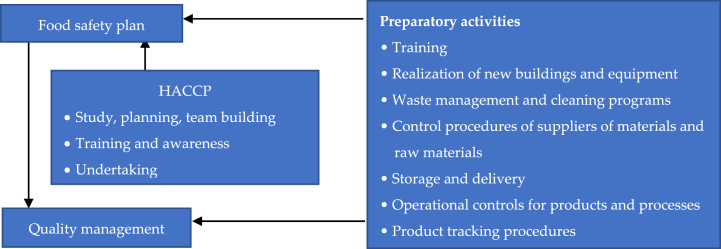
Quality management control.

The food safety must be approached in a systemic way, by applying the seven basic principles of HACCP: (1) conduct a hazard analysis, (2) identify critical points, (3) establish control limits, (4) develop monitoring procedures, (5) establish corrective actions, (6) establish audit procedures, and (7) keep records [[19], [23]]. The HACCP method forms the basis for various food safety management systems such as IFS (International Featured Standard - Food), BRC (British Retail Consortium - global food safety standard), ISO 22000 (International Standards Organization - Food Safety Management Systems - Requirements), GFSI (Global Food Safety Initiative). Based on the HACCP principles, but also on prerequisite programs included in the food safety plan, the companies have developed their own QMS (Fig. 1) [24]:

Food safety requires the establishment of a long-term management strategy, through which connections are established between all existing quality management systems in the organization. The development of a QMS requires staff training, rethinking the way activities are carried out (from planning them to checking the final result - the food product), awareness of the impact that each action has on the quality of the food product. Thus, QMS encompasses elements relating to the organizational structure (determining the staff training needs, from manager level to worker level; redefining the organization chart, reviewing job descriptions), the way processes are carried out (with a presentation of specific activities and the duties of each person involved), process auditing, etc. QMS should be seen at organizational level, as a concept designed to enable the continuous development of the company, to exude confidence in relations with business partners and consumers, to demonstrate the company's ability to provide quality products. QMS is a proof of compliance with quality standards.

### 2.2 Food quality and safety management

Food safety management systems improve several production processes, including HACCP implementation, health and safety procedures, supplier management and traceability [25,26]. Implementation of food safety management systems is associated with improved food quality and safety, and that effective implementation requires the integration of multiple disciplines and stakeholders. Food safety management systems are most effective when they are tailored to the specific needs of each organization, and that the most successful systems incorporate a combination of formalized processes and technology-assisted monitoring. The implementation of food safety management systems is associated with a reduction in the number of foodborne illness outbreaks and other incidents of food contamination. The presence of a food safety management system is associated with improved food safety through better food handling practices, better environmental monitoring and improved risk communication [27].

Food safety management systems can enable food production organizations to increase their profitability and competitiveness. Implementation of food safety management systems is also associated with increased employee productivity [28], customer satisfaction [29] and decreased allergic reactions from food [30]. Other studies associate implementation of food safety management systems with improved environmental quality [31].

The International Organization for Standardization (ISO) was founded in 1947. More than 19,000 international standards geared toward businesses and technological groups have been published by it thus far. The standards published by ISO serve as the foundation for the quality and food safety management systems. These standards are created using a complicated process that combines the extensive information and experience amassed by scholars from throughout the globe. By putting the relevant international standards into practice, the food business becomes more productive and the barriers to the global market are eliminated. Additionally, conformity with international standards provides buyers with additional assurance that the products are secure, effective, and environment-friendly. Utilizing ISO provides many advantages to organizations, including lowering manufacturing costs, improving efficiency operations and optimizing production lines, boosting customer satisfaction and quality, expanding, preventing the emergence of trade barriers, facilitating access to international markets, boosting market share, boosting productivity and an organization's competitive advantage, developing ecological advantages, and preserving the environment.

The British standard BS 5750, which was introduced in 1979 and stands for the quality assurance standard based on the NATO series of standards, particularly AQAPS, is the foundation for the ISO family of standards. The family of ISO 9000 standards, which includes the most well-known ISO standards, takes a diverse approach to various aspects of quality management. Organizations which seek to guarantee that their services and products always fulfill client needs and that the quality assurance system is updated on a regular basis can use these standards as a set of recommendations and tools.

The level of knowledge of quality assurance systems can be evaluated from at least two perspectives: identifying the number of organizations, companies, institutions and factories that have implemented such management systems and identifying in their framework of the staff's level of knowledge of the requirements imposed by them. Companies without certification might nevertheless use and apply quality assurance standards. Yet in recent years, clients have become more demanding and one of the first requirements they impose when choosing suppliers is the presence of a certificate attesting to the fulfillment of the requirements set forth by the quality assurance standards. The effectiveness of a food quality and/or safety assurance system depends on the individuals within the businesses that have adopted and/or certified it.

The existence of a very large number of distinct food safety standards, which food suppliers must adhere to and certify in order to be able to bring their products to the market, is exerting pressure on the global food business. The rising costs of certification in conjunction with the strict private standard requirements suggest an increase in costs without improving food safety. As a solution to these issues, ISO has created a solid standard that will be acknowledged and backed by global consensus, creating good practice norms globally.

With the title “Food safety management systems - the requirements for any organization in the food chain,” ISO 22000 was released in September 2005. The Food and Agricultural Organization (FAO) and the World Health Organization (WHO), which established the Codex Alimentarius Commission, also made contributions to the creation of this standard. Even today, the HACCP (Hazard Analysis and Critical Control Point) codex is recognized as a crucial management tool for managing food safety, especially when used in conjunction with an auditable management system. In reality, ISO 22000 will make it simpler for businesses all over the world to implement it in a way that is unique to each product or nation.

Over the last 10 years, scientists have made several significant discoveries related to quality management in the food industry. There have been significant advancements in food safety technology, including the development of rapid testing methods and the use of blockchain to track the origin and movement of food products. These technologies have helped to reduce the risk of foodborne illness and improve the traceability of food products. Dr. Aliyar Fouladkhah and his team developed a rapid testing method to detect salmonella in food products, which can provide results in as little as 24 h [32]. Other researchers have been studying the use of infrared spectroscopy to identify contaminants in food products, which can help to improve food safety [33,34].

Scientists have recognized the importance of effective supply chain management in ensuring food safety and quality. This includes identifying potential risks in the supply chain and implementing measures to mitigate those risks. Dr. John Spink and his team have been studying food fraud and the risks it poses to the food supply chain [35]. Their research has highlighted the importance of implementing measures to prevent food fraud and ensure the safety and quality of food products. Other studies have been researching the use of blockchain technology to track the movement of food products and reduce the risk of food fraud [36].

Researchers have explored the use of artificial intelligence (AI) in the food industry to improve quality control and reduce waste [37]. For example, AI can be used to monitor food production processes and identify potential quality issues [38]. AI enables automated monitoring and detection of potential hazards, proactive risk assessment and prediction, real-time decision support, and improved traceability in the food supply chain. By analyzing large volumes of data from various sources, AI systems can identify patterns, deviations, and potential risks, allowing for early intervention and prevention of foodborne illnesses. AI-driven technologies enable more efficient and accurate monitoring of critical control points, reduce reliance on manual inspections, and enhance overall food safety measures.

The implementation of quality management systems, such as HACCP, has become increasingly important in the food industry. These systems help to ensure that food products are safe and of high quality. It is stated that the implementation of Hazard Analysis and Critical Control Points (HACCP) in small-scale food processing facilities can help to improve the safety and quality of food products [39]. Also, the use of microbiological testing to ensure the safety and quality of food products is an important component of many quality management systems [40].

There has been a growing focus on sustainability in the food industry, including the use of sustainable farming practices and the reduction of food waste. These efforts can improve the quality of food products and help to protect the environment. Dr. Adrian Muller and his team have been researching the environmental impacts of food production and consumption, and have highlighted the need for more sustainable farming practices [41]. Dr. Shauna Downs and her team have been researching the use of traditional farming practices to promote sustainability and improve the quality of food products [42].

### 2.3 HACCP and food quality management

In past years, food safety issues have occurred often, threatening the health and safety of people's livelihoods and lives. The notion of “from farm to table” has been promoted, but the most pressing issue for the government, the related businesses, and the authorities is still how to implement an effective food safety management system. HACCP is a new form of economic regulation or intervention. It's a private process management tool. Now it's mandatory for whole sectors, requiring specific risk assessment to relate HACCP regulations to public health results. Food safety requirements under obligatory HACCP are implicitly defined by risk assessment [43]. HACCP is a methodical preventative approach to food safety and allergic, chemical, and biological hazards in production processes that might result in an unhealthy final product, and it includes measures to decrease these risks to an acceptable level [44].

The HACCP concept was first developed in the 1960s by the U.S. National Aeronautics and Space Administration (NASA), working with Pillsbury, one of the world's largest producers and cake manufacturers of cereals and other food products, to ensure crumb- and pathogen-free food that had extensive shelf-life properties for space travel—the first pathogen monitoring and measurement requirement imposed on the food industry [45]. Despite the fact that the initial HACCP plan only comprised of three principles as compared to the seven principles that are currently in use, the execution of this program was able to decrease the risk that is associated with foodborne pathogens in food. Over time, as Pillsbury began to get more involved, the business also began incorporating aspects of the system into the food safety procedures used internally. After the publication of a study on HACCP in 1980 by the World Health Organization (WHO) and the International Commission on the Microbiological Safety of Foods (ICMSF), WHO Europe endorsed its implementation in 1983 [46]. The seven key concepts that are central to HACCP were presented for the first time in 1992. During this time, HACCP found support in a variety of food safety meetings and groups, one of which was the Codex Alimentarius Commission, which in 1993 adopted guidelines for the application of the HACCP system.

HACCP's efficiency in avoiding food-borne illnesses and reducing food safety hazards depends on proper implementation and utilization [2]. Food companies that adopt HACCP must assess its performance and implementation [47]. To do so, analyze the impact of HACCP's obstacles. Firms must acknowledge the particular difficulties they face at each step of HACCP and develop appropriate intervention approaches. The implementation of a HACCP system reduces costs associated with production and increases customer satisfaction [48]. Implementing HACCP-based processes increases speed and accuracy in manufacturing operations. HACCP-based systems are effective in preventing foodborne illnesses and the implementation of such systems creates a climate of increased safety. Implementation of HACCP-based systems is associated with improved food safety through better food handling practices, better environmental monitoring and improved risk communication. A combination of HACCP-based control strategies, including sanitary and physical controls, are necessary for optimal food safety [49]. The successful implementation of HACCP-based systems depends on appropriate training and monitoring mechanisms [50]. Implementing a HACCP-based system requires organizations to address food safety from a holistic approach and recognize the interconnectedness of all factors involved in the food system [51].

Overall, these and other discoveries have helped to improve quality management in the food industry, making food safer, more traceable, and more sustainable over the last decade. Their research has helped to improve the safety, quality, and sustainability of food products, and will continue to shape the future of the food industry.

### 2.4 The food safety management system - selection criterion in public procurement

The fact that companies implement food safety systems increases the confidence of their customers (either individuals or businesses) in the food they sell. The consumers are interested in safe, verified products that do not harm their health. Every year, each country budgets significant amounts of money for the purchase of food to supply establishments where food is needed, such as the hospitals for their patients, the educational institutions (such as universities, high schools, kindergartens where students, pupils and pre-school children are present), the military units, the institutions responsible for the social care of people, etc.

The main problem of public institutions in the process of purchasing food is the insufficient level of knowledge to select the most viable supplier [52]. An important criterion for selecting the food suppliers is compliance with the HACCP principles. The contracting authority establishes such a requirement on the basis of the provisions of the Directive No 43/1993 that aims to determine the general rules of food hygiene and the means of verifying compliance. The rules of this directive aim to increase the consumer confidence in food safety, based on the ability of firms to produce, package, transport, and market safe food [53].

At the EU level, the Directive No 24/2014 (Art. 62) entitles the contracting authorities to require economic operators, through contract award notices, to submit the certificates issued by the independent bodies attesting compliance with food quality assurance standards [54]. The contracting authorities are obliged to recognize equivalent certificates issued by the independent bodies located in other Member States during the evaluation of tenders.

The tender compliance with all the conditions of the procurement documentation is a prerequisite for consideration [55]. If a tenderer has not obtained the certificate within the deadline for reasons beyond its control, further evidence may be submitted attesting that the applied quality assurance measures comply with the relevant standards. In this respect, the surveillance audit report, drawn up by the independent body, may be submitted to prove the ownership and maintenance of the food safety management system.

Such a report, in which the auditors have recorded that the company complies with the quality management rules, constitutes an equivalent document within the meaning of Article 62 of the Directive 24/2016 [54]. The fact that the quality certificate has not been issued by the time the tenders are submitted cannot represent a reason for rejecting tenders, as long as there is a successfully completed surveillance audit.

On the other hand, the requirement to present a particular certification, such as ISO 22000 [56], constitutes a restrictive measure, limiting the access of economic operators to the tendering procedure. In such a situation, only those companies that are ISO 22000 certified shall be able to submit a competitive tender and those that have implemented and maintain another quality system, whatever its name, shall not be accepted. It is in the interest of the contracting authority that the future supplier has a management system that ensures food quality [57]. Opening up the procurement procedure to more competition shall be achieved by accepting standards equivalent to the one set by the contracting authority. To this end, in the tender notice, the qualification requirement shall include also alongside the title of the standard the mention “or equivalent”, such as “submission of ISO 22000 certificate or equivalent” [58].

The quality standards implementation based on the HACCP principles, in addition to achieving food safety and customer confidence in the company concerned, allows access for the companies to public tenders and contributes in the same way to the increase of the customer portfolio.

### 2.5 Barriers and opportunities for implementing HACCP

HACCP is an important tool for businesses operating internationally, helping them to what can go wrong in food production. Regarding the implementation of HACCP, businesses face both barriers and opportunities when they want to implement such a system.

The literature was researched to identify the main barriers, and a selection of articles presenting the challenges faced by various developing and developed countries was performed. The analysis of the main barriers began by searching for relevant articles with the help of Scopus and Google Scholar databases from 2000 to 2021. Only articles in English were selected from the databases used. Therefore, 13 articles considered relevant for the field of study have been analyzed. To identify the most suitable articles, the search included the following keywords: barriers and opportunities, HACCP, difficulties in HACCP implementation, benefits in HACCP implementation, and HACCP implementation. The results for the key barriers for implementing HACCP are shown in Table 1 and the results for the main opportunities for implementing HACCP are presented in Table 2.

**Table 1:** Barriers for implementing HACCP.

Barrier category	Barriers	World region	Short description of barrier	Methodology used	References
**Financial**	High costs for restructuring the organization	Armenia	For enterprises, especially for medium and small ones, the implementation of HACCP represents a significant financial effort.	Qualitative and quantitative research. The studies carried out by other companies were analyzed, focus groups and survey of economic agents.	[59]
**Financial**	Financial constraints	Canada	Companies do not have enough resources to invest in such an initiative.	Quantitative research. A structured questionnaire was created with the main benefits, costs and barriers based on the specialized literature in the food field. 1044 food manufacturing companies in Ontario participated in the survey.	[60]
**Organizational**	The extent of change		The change is a big one and will not be accepted by all employees.		
	Low priority		Other investments are more important at the moment for some companies		
**Knowledge**	No previous exposure to HACCP principles	Philippine	The inability of employees to adapt to HACCP principles and requirements	Survey of small and medium-sized food companies.	[61]
**Financial**	High costs for implementing HACCP	UK	Plans for HACCP implementation must be carried out by an external organization, which entails additional costs.	An in-depth qualitative research based on narrative interview was carried out.	[62]
**Bureaucracy**	Long time to complete the documents		It has been proven that the documentation that needs to be prepared is a burden for organizations, and the companies that deal with the creation of the documents, do not give priority.		
**Financial**	Lack of financial resources	Zimbabwe	Most companies face financial problems that do not allow them to implement HACCP.	The research was carried out through a structured questionnaire distributed to specialized companies	[63]
**Financial**	Financial constraints	Turkey	Companies do not feel sufficiently prepared from a financial point of view, as great financial efforts must be made to implement HACCP.	A survey based on a questionnaire was carried out, where 115 enterprises from the food sector participated.	[64]
**Knowledge**	Lack of knowledge		Business revenues do not allow investment in such initiatives		
**Bureaucracy**	Lack of involvement from the authorities		The authorities do not provide enough support for businesses to implement HACCP		
**Motivation**	Lack of employee motivation		Employees do not feel sufficiently motivated to acquire the necessary HACCP knowledge		
**Financial**	Insufficient funds implementation	Greece	Businesses are not financially prepared for HACCP implementation	Qualitative research was carried out by analysing the specialized literature	[13]
**Organizational**	Lack of commitment for implementation		The enterprise management does not give importance to the implementation of HACCP. Employees are also sceptical of this approach.		
**Knowledge**	Insufficient knowledge and skills	Algeria	Both managers and employees do not feel ready for this approach, considering that they lack the necessary knowledge and skills.	The data were collected with the help of 46 companies in the field. The study was based on quantitative research using a questionnaire.	[65]
**Financial**	Lack of funds		Companies do not have enough money to invest in HACCP implementation.		
**Motivation**	Lack of desire		Employees and managers have become accustomed to the old ways of working, monotony, and routine set in the workplace. Thus, they lack the motivation to learn something new. Also, the long-time span for implementation demotivates them.

**Table 2:** Opportunities for implementing HACCP.

Opportunity category	Opportunities	World region	Short description of opportunity	Methodology used	References
Safety	Reducing the level of pathogens	Netherlands	Food safety is improved, eliminating the risks that can cause health problems for the consumer. This increases consumer confidence and increases the company's competitiveness in the market.	Qualitative research by analysing specialized literature.	[66]
Development	Attracting new customers	Greece	The company's ability to retain old customers and attract new ones is enhanced. Also, the company allows itself to enter new markets and increase the prices of the products, so that they are related to the quality, without losing customers.	Quantitative research in the form of a questionnaire was carried out at the food companies of the “ICAP” group. 91 companies participated in the study out of the 106 to which the questionnaire was sent.	[67]
Safety	Reduction of microbes in food		Products are improved by reducing microbes and pathogens. Extending the shelf life for products.		
Organizational	Improving procedures		A major benefit is represented by the improvement of internal procedures that allow the creation of quality products at low costs.		
Organizational	Improving internal procedures	Italy	Information manages to circulate within the company much more efficiently by creating an internal communication network between management and employees. HACCP implementation comes with new organizational solutions that improve food quality. Managers have seen a decrease in corrective actions, resulting in cost reduction.	Quantitative research was carried out based on a questionnaire at the level of managers of meat and dairy producers.	[68]
Organizational	Improving management	China	Within the companies that have implemented HACCP, an improvement in management within the organization has been observed. Managers have begun to pay more attention to their relationships with employees and suppliers, which leads to increased competitiveness and improved food safety.	The study was conducted in Pudong, a rural region heavily populated by food manufacturing companies. It was based on the distribution of a questionnaire to 250 companies in the area.	[69]
Safety	Increased product safety	Serbia	It increases the company's ability to offer quality and safe products for the health of consumers. Thus, the confidence of the buyer increases, and the number of complaints decreases.	A quantitative survey based on a questionnaire was conducted among 77 meat producers in Serbia.	[70]
Financial	Increase in product prices		Considering the quality of the products, companies believe that they can have financial increases by increasing the prices of the products offered. Their price must reflect the quality, and consumers are willing to pay more to receive safe products. Production costs are reduced, so the company can invest in something else.		
Organizational	Discipline at work		Employees are responsible and disciplined at work, being aware of the importance of their work to provide the safest food.		
Development	The ability to enter new markets		The company's chances of entering other markets increase considerably.		
Safety	Improving product safety	Algeria	Food protection steps are improved, and microbes are limited. This system allows the workers to carry out their activities in a clean and disinfected environment.	46 companies that implemented HACCP were involved in the study using a survey based on a questionnaire.	[65]

Despite the benefits brought by HACCP for the management system of a company active in the food sector, analysing the research listed above, we note that the biggest barrier encountered by economic agents is represented by budgetary constraints, as investments are needed for restructuring the organization and implementation of the new system. Integrating HACCP into the fabric of a business requires ongoing funding for employee training, salary increases, employee training, and hiring of skilled labour.

Another barrier, mentioned very often in the literature refers to the knowledge and perception of employees, regarding the implementation of HACCP. There is a lack of motivation on the part of the management, but also of the employees. None of the study participants went through a training program regarding HACCP implementation. It was noted that the organizations participating in the study consider their products safe, and they do not require the implementation of such a plan. Thus, considering that their products are safe, they do not want to accumulate new knowledge to approach HACCP.

All the documentation required for implementation and the lack of support from the authorities are other barriers. They are of the opinion that the existence of specialized training programs and support through funds from the authorities is necessary. Also, the implementation requires numerous studies and documents that are carried out by a third party, which according to the companies, complicates the process and makes it more difficult.

After analysing the studies carried out within the companies that have implemented HACCP, or intend to do so in the future, a series of opportunities have been extracted, which can allow the business to develop.

Among the most listed opportunities were those related to food safety and organizational improvements. The implementation of the HACCP system allows the company to take a more careful approach to food safety, identifying potential hazards at any stage of production. In the framework of HACCP, the dangers (chemical, biological or physical) that may exist in food from the moment of handling and manufacture to the distribution and consumption of the product by the buyer, are evaluated. This improvement comes with a number of benefits such as: retaining customers, attracting new customers, limiting complaints, increasing consumer confidence, and maintaining market access.

This new system comes with organizational changes from top management to employees. HACCP provides the perfect framework within the company where employees work happily for the success of the business.

Even though the high implementation costs are always mentioned, the financial opportunities that appear with the implementation of the HACCP system were also highlighted. Its implementation allows the company to increase product prices, lower production costs, eliminate errors and reduce complaints, thus eliminating part of the expenses. Customer trust increases for companies that have implemented such a system, choosing their products because they are safe, thus revenues are increasing.

The implementation of HACCP is an essential component for reducing risks and increasing the quality of products offered to consumers. We believe that there should be more research highlighting the importance and benefits of implementing such a system. Also, at the government level, it is essential to have training and information campaigns for companies in the food industry. It is necessary to involve governments by highlighting the problems encountered by companies that have not Implemented such a system. Thus, consumers will turn to secure companies, and companies will focus on implementing such a system to attract customers.

The main barrier to the implementation of HACCP is the financial constraint, especially for small and medium-sized companies that cannot afford such a financial effort, the existence of a source of financing and consultancy is necessary.

## 3 Materials and methods

Scientific production evaluation provides the foundation for many significant decisions, including organizational development, university funding priorities, legislation development, cooperation opportunities, and many others [18]. The impact of a publication as a criterion of research quality becomes essential nowadays. A subfield of scientometrics defined as bibliometrics uses quantitative research such as mathematical and statistical techniques to analyze the activity of scientific publications.

There are many reasons to conduct bibliometric and systematic reviews, but they are most frequently performed to provide a summary of prior research on a particular subject which may be used to investigate, assess, and synthesize current research, investigate at specific research issues, and suggest future research directions. The methods employed to perform reviews have evolved to accomplish a variety of objectives as scientific literature has expanded and multiplied in the electronic era. In the current digital world, scientific production is being created at a faster pace, that generates difficulties for academics, regulators, researchers, and professionals to stay current with developments in their fields [71].

The interest in bibliometric analysis as a significant scientific endeavor has grown significantly in recent years. Various study domains, such as: agronomy [72], business [73], environmental sciences [74], economics [[75], [76], [77]], food science technology [78], green sustainable science technology [18,79], management [80] have successfully implemented bibliometrics in food or business industry.

In order to establish correlations using social network analysis to obtain a broad view of the research area and using keyword network assessment to find the most crucial topics and the relationships between them [81], bibliometric analysis has two main applications: the first is descriptive, which involves evaluating the performance of journals, authors, institutions, and countries; the second includes determining correlations using keyword network approach to determine the most crucial topics and the connections among them.

Through the use of bibliometric analysis, the current study aims to assess how HACCP influences modern quality management systems in the food industry. In consideration of this, the article's aims are to present a critical overview of prior research, identify trends and patterns in HACCP and modern quality management systems in the food industry research, and highlight the most important related topics and study gaps using a comprehensive bibliometric analysis. By rigorously identifying and evaluating the scientific production, major contributions to the field, and major future research directions, the results show the structure, evolution, and important trends and implications of the HACCP and modern quality management systems in the food industry research field. Moreover, through an in-depth systematic review, this research provides a thorough evaluation of barriers and opportunities of implementing HACCP as modern quality management system in food industry organizations.

Since it encourages rigorous planning, consistency in execution, and transparency that enables study replication, a protocol is crucial for bibliometric analysis, as it enables researchers to anticipate difficulties, obviate arbitrary choices, uphold study integrity and responsibility [18]. There are various protocols and research frameworks presented in the literature that can be used in bibliometric analysis, including the bibliometric protocol [82,83], bibliometric analysis toolbox [84], PRISMA flow diagram [85,86], PRISMA-P [87], research design of bibliometric analysis [88] and various other adaptations of these. The PRISMA statement is developed to assist in reporting a systematic review with meta-analyses, but it can also be easily applied to other types of review [89]. After analyzing the data from the aforementioned sources and creating the specified frames to meet the goals of the current research, the authors modified the bibliometric technique to include the bibliometric analysis as well as the systematic review of barriers and opportunities for implementing HACCP in food industry organizations (Appendix A).

The research protocol combining bibliometric analysis and systematic review (Appendix A) contains three main steps [71]. Step I is initiated by formulating the research questions based on the primary goal of this study, the subject under investigation, the literature review and the peculiarities of bibliometrics and systematic review as research methodology. The research questions were amply presented in the Introduction section. After formulating the research questions, the research protocol was established, containing bibliometric review protocol and systematic review based on PRISMA statement. Scopus was chosen as the database for data set since it is one of the most established bibliographic data sources [90] and it focuses on comprehensiveness [91]. Furthermore, Scopus has established itself as a trustworthy and, in some ways, even superior to Web of Science source of bibliographic data [92,93].

The Scopus keyword search included the following keywords: HACCP”, “Food”, “quality” “management” AND „system” in the “Article title, Abstract, Keywords” section of the database. The search was conducted on January 02, 2023, and 362 papers were obtained. One document was early access, published in 2023 and it was eliminated from the list. Therefore, the publication data set included 361 papers (Table 3).

Step 2 of the research protocol entails carrying out the review procedure using the subsequent methods:
- For the systematic review: synthetizing the barriers and the opportunities of implementing HACCP in organizations.
- For the bibliometric review implied applying the following techniques [71,82]:
1. Descriptive statistics - examines the growth of annual scientific output, the spread of publications by document type, subjects of study, highly prolific academics, highly productive journals, and the dissemination of scientific production by countries and funding agencies.
2. Performance analysis - which studies whether scientific production has evolved in connection to the citations acquired for the studied works.
3. Science mapping, performed using VOSviewer program (version 1.6.18, Leiden University, The Netherlands) and bibliometrix R-package, it encompasses three subtopics: conceptual structure: co-word analysis (It is carried out to identify prominent authors and key phrases related to a study topic. Analyzing the changing theme progression through time is also important to spot emerging and outdated issues [83]; intellectual structure: co-citation analysis (It is used to detect patterns in concepts among publication and cluster texts into multiple topics based on its conceptual structure. It serves as the foundation for the semantic clustering of related documents within the same field [83] and social structure: co-author analysis.

**Table 3:** Scopus Keywords search results (January 02, 2023).

Combination of Words	Research Results
HACCP	3292
HACCP AND Food	2557
HACCP AND Food AND quality	1040
HACCP AND Food AND quality AND management	459
HACCP AND Food AND quality AND management AND system	362
Total (after removing early access articles with publication year 2023)	361

The systematic review implied Scopus keyword search using the following keywords: “Food”, “HACCP”, “quality management system” AND “barriers” in the “All fields” part of the database.

In order to create and visualize bibliometric networks based on connections amongst co-citations, co-authorships, or bibliographic coupling using the visualization of similarity (VOS) mapping technique VOSviewer was utilized [94]. It also features text mining capabilities for constructing co-occurrence networks of significant words and phrases gleaned from various scientific papers [95]. Furthermore, it calculates the overall link strength to measure how many publications contain two or more of the indicators (keywords, authors, citations, etc.) [82].

For many units of analysis, including researchers, research institutes, and countries, co-authorship networks can be built. Equation (1) indicates the mechanism to construct co-authorship networks, although this can be applied to other units of analysis in VOS [94,96]:

(1) $$r_{i}=\sum_{i=1}^{R}a_{ik}$$

Where $r_{i}$ represents the number of researchers for publication *i, R* stands for the number of researchers who are included in the analysis and $a_{ik}$ denotes the researchers × publications matrix.

The association strength is a method that VOS uses to normalize co-occurrence frequencies. The association strength $s_{ij}$ is computed as follows [93] (Eq. (2)):

(2) $$s_{ij}=\frac{{at}_{ij}}{t_{ii}t_{jj}}$$

Where $at_{ij}$ represents the number of total abstracts where topic *i* and *j* appear together. $t_{ii}$ and $t_{jj}$ count the instances in which topics *i* and *j* appear together in the abstracts.

The relationship $r_{ij}$ between two items *i* and *j* is calculated using the association strength as follows [97] (Eq. (3)):

(3) $$r_{ij}=\frac{o_{ij}}{x_{i}x_{j}}$$

where $o_{ij}$ indicates the number of co-occurrences of items *i* and *j* and $x_{i}$ and $x_{j}$ stand for the total number of occurrences of items *i* and *j*.

The VOS mapping approach creates a map based on the similarity matrix after computing the similarities. Let n represent how many objects need to be mapped. The VOS mapping algorithm creates a two-dimensional map in which the items are positioned so that the distance between any two items, *i* and *j*, as accurately reflects their relationship $r_{ij}$ as possible. Objects with a high degree of resemblance should be placed close together, whilst items with a low degree of similarity should be placed apart [93]. A conceptual structure map for performing quantitative bibliometric and scientometric research was generated using the bibliometrix R-package also (http://www.bibliometrix.org).

The analyses of the final results of bibliometric and systematic review, presentation of research findings, namely a thorough content analysis and evaluation of scientific production, are included in Step 3 of the research methodology. Furthermore, trends, possible areas of study and study limitations are identified and presented.

## 4 Results

The main results obtained after the centralization of the data through Bibliometrix from the R program are presented in Table 4. Thus, the 361 documents on the subject of HACCP and modern quality management systems in the food industry were published between 1978 and 2022, generating an annual growth rate of 4.16%. Each document recorded an average of approximately 13 citations, and in total the 361 documents included 12374 references. In total, there are 983 authors who approached the topic of analysis through this research, of which 103 documents have a single author, but on average there are approximately three co-authors per document. The percentage of international co-authorships for this topic is 13.02.

**Table 4:** Synthesis of data analyzed with Bibliometrix.

Description	Results
Timespan	1978–2022
Documents	361
Annual Growth Rate %	4,16
Document Average Age	11,4
Average citations per doc	13,06
References	12374
Keywords Plus (ID)	1588
Authors	983
Single-authored docs	103
Co-Authors per Doc	3,03
International co-authorships %	13,02

**Fig. 2:**
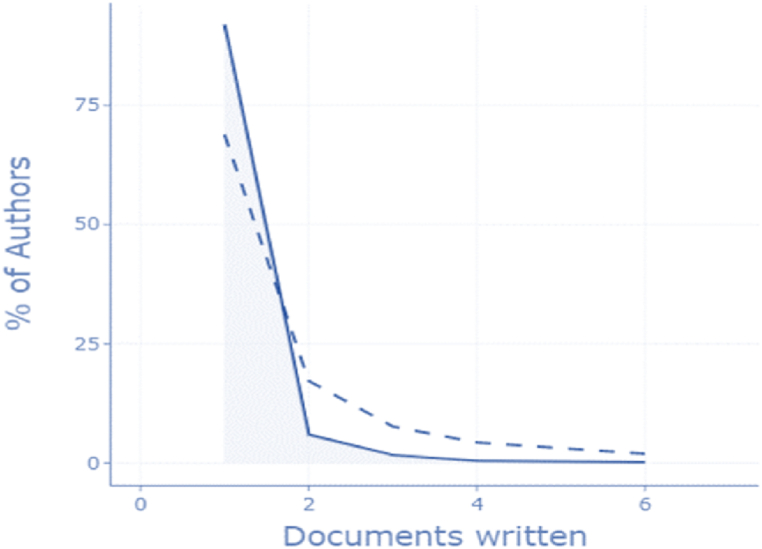
Lotka's Law based on bibliometrix Results.

Lotka's law is the first scientific or informetric rule. A bibliometric example is Lotka's rule of scientific productivity, where the number of authors was plotted against the number of contributions made by the writers on a logarithmic scale. It details the regularity of author contributions in every specific topic [98]. For the topic HACCP and modern quality management systems in food industry, according to Lotka's Law (Fig. 2, generated on Table 5) it results that most of the authors published only one document on the analyzed topic, namely 91.9% of the total authors. Only four authors published four documents and only 0.2% of the total authors (n = 2) published six articles.

**Table 5:** Lotka's law - detailed results.

Documents written	N. of Authors	Proportion of Authors
1	903	91.9%
2	58	5.9%
3	16	1.6%
4	4	0.4%
6	2	0.2%

**Fig. 3:**
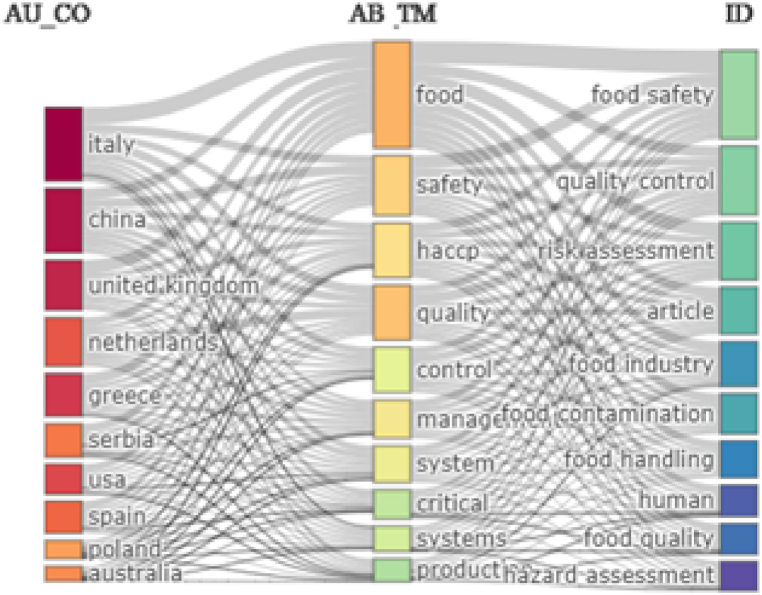
Three-Field Plot – Relationship between authors' countries, abstract words and keywords plus.

The relationships between the main 10 authors’ countries (left side), abstract words (middle) and keywords plus (right side) are depicted in Fig. 3. According to Bibliometrix, most authors that published papers on HACCP and modern quality management systems in food industry come from Italy, China, United Kingdom, Netherlands and Greece. As respects to main words included in the abstracts of the scientific production, these include: food, safety, HACCP, quality and control. Food safety, quality control and risk assessment are the main focus of the studies in the above-mentioned topic. Studies have concentrated on these relevant elements to uncover the new trends and discoveries related to HACCP and modern quality management systems in food industry.

Based on the processing of the data from the Scopus database, 361 articles were published between 1978 and 2022. Although the first two articles were published in 1978, until 1982 no other indexed document was published in Scopus on this topic. The academic community had not yet paid much attention to the investigated topic in this period. However, several ground-breaking articles created the framework for the study of HACCP and modern quality management systems. Also, until the beginning of the 1990s, there was not a large number of scientific production in the field of HACCP and modern quality management systems in the food industry. For this reason, Table 6 shows the evolution of the number of articles, citations and average citation per article for documents published in the period 1978–2022, but divided over longer periods of time, i.e., every five years, with the exception of the first period, 1978–1992 which spanned 14 years due to the small number of articles published in that period, as well as the citations recorded for the published publications. There is a clear correlation between the overall number of citations and the number of articles produced. The most citations were recorded for articles published in the last five years, resulting in an average of approximately 24 citations per article.

**Table 6:** Scopus publication years.

Years	Articles	Citations	Average citations/Article
2022–2018	74	1798	24,30
2017–2013	95	1493	15,72
2012–2008	78	904	11,59
2007–2003	63	346	5,49
2002–1998	33	120	3,64
1997–1993	13	33	2,54
1992–1978	5	16	3,20
**Total**	361	4710	13,05

**Fig. 4:**
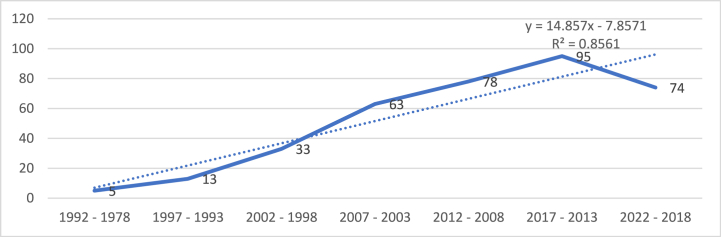
Articles' evolution over time.

The number of publications represents a leading determinant of a field's development base and a guideline to forecasting developments. Based on an examination of the distribution of yearly scientific production, Fig. 4 illustrates the major developments of research in the area of HACCP and modern quality management systems in food industry. The graph also includes the linear trendline, highlighting a relatively constant increase in the number of articles published during the analyzed period, except for the last five years, in which scientific production slightly decreased. Furthermore, the correlation between the variables is calculated using the R squared equation. The reliability of a trendline is measured by its R-squared value; the closer $R^{2}$ is to 1, the better the trendline matches the data. For the information collected from Scopus database, $R^{2}$ is calculated and displayed in Fig. 4, having a value of 0,8561, which is close to 1 and the trendline fits the data very well.

**Fig. 5:**
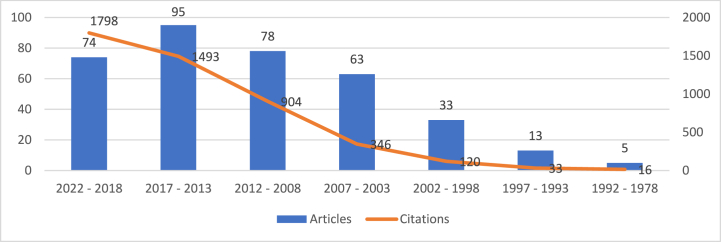
Citation evolution analysis.

We identified that the majority of the most-cited publications in the topic of HACCP and modern quality management systems in food industry were published in the last five years, namely during 2018–2022, indicating that the research findings during this time period were more important and relevant to other scholars. We did this by examining the local and worldwide citation rates of the articles in this field. Fig. 5 illustrates the trend of citation in relation to the number of published documents in the same periods of time. It is found that this field is one in continuous evolution, which indicates that the scientific production must be up to date in order to obtain citations and be taken into consideration by other researchers. Research older than 10 years seems to be no longer relevant and is mostly taken into account in literature review type of works.

**Fig. 6:**
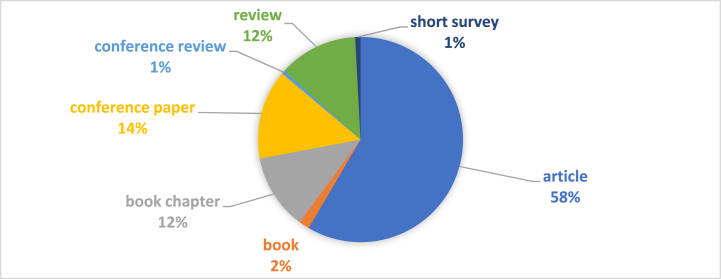
Bibliometric analysis - cocument types.

Regarding the document types published in the field of HACCP and modern quality management systems in the food industry, it is found that most of them are articles, respectively 58% of the total scientific production. These are followed by conference papers (n = 14%) and reviews and book chapters, each with a proportion of 12% of the total. The conclusion that derives from Fig. 6, in which the results of this analysis are illustrated, is that the researchers investigated the field much more carefully, having a well-developed methodology. Conference papers or book chapters are based on more theory than practical analysis of the field, and the fact that their number is reduced compared to articles published in journals reveals the emphasis placed by researchers on practical analysis.

The fourth research question aims to investigate the present status of international collaboration on HACCP and modern quality management systems in the food industry. In order to achieve this objective, a series of analyzes were performed based on information processed from Scopus, Biblioshiny, as well as a series of elements taken from Google Scholar, in the case of authors and journals that have public profiles on this platform.

A first analysis concerned the top 10 prolific authors with scientific production on HACCP and modern quality management systems in food industry (Table 7). The values included with * in the table are taken from Biblioshiny software, as the authors do not have a public Google Scholar profile. It seems challenging to directly measure or evaluate the quality of publications. Nevertheless, a number of factors can be used as a metric for published impact or quality, including the total number of citations received, the average number of citations per article, the Hirsch index (h-index), the proportion of highly cited papers, and the impact factor (IF) of journals. To evaluate the productivity and citation effect of academics, the H-index was created. Furthermore, the h-index is now being used to evaluate the production and influence of research universities and countries [99].

**Table 7:** Top Prolific Authors (Source: Biblioshiny and authors).

Author	H index	i-10 index	TC	NP on topic	PY start	Country	Institution
Luning PA	54	126	10808	6	2004	The Netherlands	Wageningen University
Jacxsens L	4*	4*	190*	4	2009	Belgium	Ghent University
Drosinos EH	49	118	7588	4	2007	Greece	Agricultural University of Athens
Dzwolak W	3*	3*	60*	3	2014	Poland	University of Warmia and Mazury
Jongen WMF	3*	3*	67*	3	2004	The Netherlands	Wageningen University
UyttendaelE M	75	284	19265	3	2009	Belgium	Ghent University
Van Der Spiegel	16	17	1593	3	2004	The Netherlands	Wageningen Food Safety Research
Worsfold D	3*	3*	52*	3	2001	UK	University of Wales Institute
Ababouch L	2*	2*	9*	2	2004	Italy	Food and Agriculture Organisation
Adams CE	2*	2*	10*	2	1994	USA	Michigan State University

TC = total citations, NP = number of publications, PY = publication year.

The most significant author in terms of articles produced is Luning PA from Wageningen University (The Netherlands). During the 18-year period, Luning wrote six publications on this topic, getting more than 10,800 citations for the overall scientific production. The H index from the Google Scholar is 54 and i-10 index is 126. Pieternel Luning and her group researched various themes such as culturally framed food safety management systems [100], shortcomings and potential remedial measures of performance of food safety management systems in African food processing firms [101], effectiveness of a HACCP-based food safety management system in Japanese milk processing facilities in a semi-quantitative manner [102], increasing food quality in the bread industry [103], or performance measuring tools and their application to food quality systems [104]. The second most prolific authors in terms of documents published (n = 4), are Jacxsens L from Ghent University (Belgium) and Drosinos EH from Agricultural University of Athens (Greece). Among the main research topics of Jacxsens in the 13 years of scientific production on the analyzed theme are food safety effectiveness assessment of management systems' microbiological performance [105] or effectiveness of a HACCP-based food safety management system in Japanese milk processing facilities [102]. Drosinos' main research topics targets techno-managerial aspects of food enterprises’ food safety management systems [106], or factors that affect the adoption of HACCP in the food industry [107].

It is important to highlight that the majority of the authors in Table 7 are from European countries, mostly from The Netherlands and Belgium. Only one author that published two articles on HACCP and modern quality management systems in food industry in from the USA (Michigan State University), namely Adams CE.

**Fig. 7:**
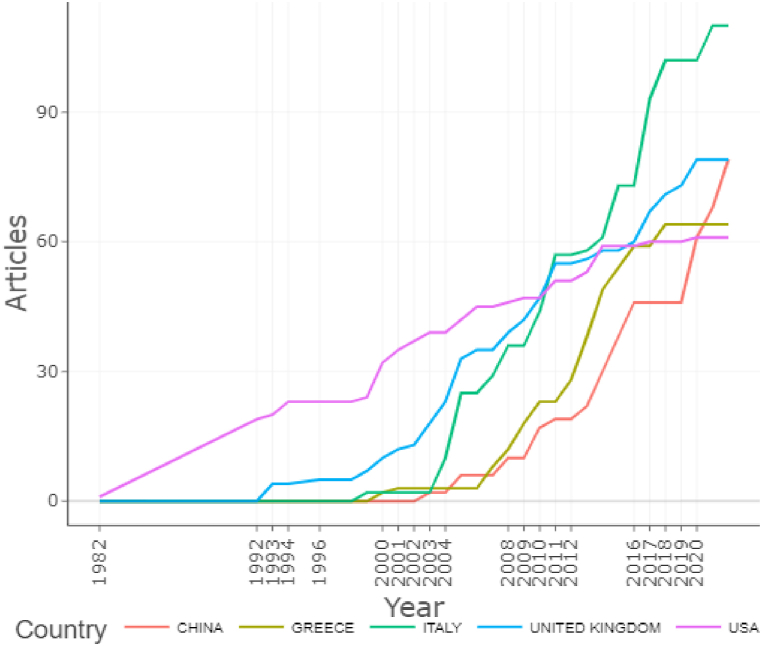
Countries' Production over Time (*Source*: Generated by authors on Biblioshiny based on Scopus Database).

The next step of the research analyzes the countries’ production over time illustrated in Fig. 7. The results generated by Biblioshiny based on Scopus database indicate that studies on the analyzed topic began in the USA, and between 1982 and 2008 it was the most prolific state in terms of scientific production. However, the research from the USA started to decrease starting from 2012, the period from which the researches of specialists from Italy, the United Kingdom and China intensified. Also, Greece had a prolific period of publications in the field between 2004 and 2017. Currently, the most prolific country, according to the data processed by bibliometrix, is Italy.

Regarding the most prolific corresponding authors' country, the United Kingdom (UK) stands out as the country from which 90 articles with corresponding authors were published. UK is also the country with the most single country publications (SCP). SCP are publications with all of the authors originating from the same country, and thus indicating intra-national cooperation [99]. The difference between the first ranked country as the most prolific corresponding authors' country and the others included in the top 10 is substantial. The following included in the ranking are the USA (29 articles) and Greece (21 articles). Likewise, the difference between the UK and the following countries is also significant (USA = 22 intra-national cooperations and Greece = 19 intra-national cooperations). Multiple country publications (MCP) represent the case in which the authors are from various nations, namely examples of international collaboration since they show cross-national cooperation [99]. In the case of MCP, there are not many international collaborations, the most are registered in the case of the UK and the USA, each with seven collaborations. In the case of China, Germany and Australia, there is only one international collaboration, and Spain, although it is the most prolific corresponding authors’ country, due to the number of articles published as a corresponding author, there is no intercountry collaboration publication (Table 8).

**Table 8:** Most prolific corresponding authors’ country and most cited countries.

Most Prolific Corresponding Authors' Country						Most Cited Countries		
Country	Articles	SCP	MCP	Freq	MCP_Ratio	Country	TC	AAC
United Kingdom	90	83	7	0,249	0078	Belgium	733	122,17
USA	29	22	7	0,08	0,241	United Kingdom	466	16,07
Greece	21	19	2	0,058	0095	USA	374	17,81
Italy	19	17	2	0,053	0105	Netherlands	337	25,92
China	18	17	1	0,05	0,056	Greece	312	16,42
Netherlands	17	15	2	0,047	0118	Italy	281	15,61
Poland	13	11	2	0,036	0154	China	249	14,65
Germany	13	12	1	0,036	0077	Canada	238	34,00
Spain	9	9	0	0,025	0	Australia	201	28,71
Australia	8	7	1	0,022	0125	Spain	175	21,88

SCP - Intra-country collaboration publications; MCP - intercountry collaboration publications; Freq - Frequency; TC – Total citations; AAC – Average articles citations.

The most frequently cited publications are one of the most significant markers to measure the hot issues in a professional field. Comparing articles generated with and without international collaboration, it highlights that scientific production created through international collaboration (MCP) received more citations per article than documents published by authors from a single country. This suggests that raising the number of citations requires worldwide cooperation. It is noteworthy to mention that the countries with the lowest levels of international cooperation, like Australia and Spain, had fewest citations per publication. The country with the most citations is Belgium (n = 733), also having an average number of articles citations. This is followed by UK, USA, Netherlands and Greece, each registering over 300 citations.

**Fig. 8:**
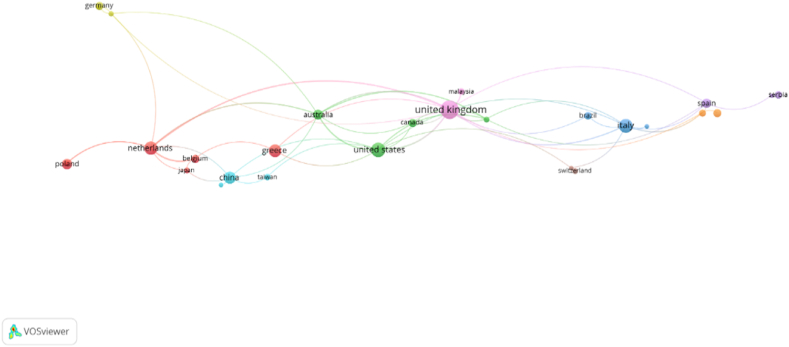
Science Mapping - Social Structure: Co-authorship analysis. – Countries Cooperation Network Graph.

To deeper analyze the international collaboration on HACCP and modern quality management systems in the food industry, a country cooperation network graph was created using VOSviewer software. This examines the most successful international relationships and illustrates a map of the co-authorship countries. The circles' size reflects the number of documents that researchers from most prolific countries have written, and the colors symbolize the working groups [82]. There were set several limitations in VOSviewer to generate the network visualization map from Fig. 8: the chosen counting technique is full counting, the maximum number of countries per document is set at 25, while the minimum papers for a country is set at 3. Imposing these limitations, of the 80 countries, 37 met the thresholds. Due to the fact that the international collaboration on this topic is not at high levels, even imposing such low limitations, 9 clusters were generated, forming 52 links and a total link strength of 70. 20 papers did not have the country or territory defined in the published documents. With significant global cooperation, the United Kingdom, The United States and The Netherlands represent most active nations on this topic. Serbia, Malaysia and Taiwan's contributions were deemed to be minimal.

Each academic publication has a distinct goal, range, concentration, and area of interest. Popular journals are a significant source of cutting-edge research content in addition to representing the preference and recognition of the majority of researchers in this discipline. Identifying articles from well-known publications can also help researchers identify their research findings with accuracy [88]. The journals were sorted in descending order based on the quantity of papers published in the field of HACCP and modern quality management systems in food industry. Most articles on the analyzed topic were published in Food Control journal, which is part of Quartile 1, has a Cite score of 9.3 in 2021 and SJR 1.083. The journal is indexed in Scopus starting from 1990 until present times. Food Control represents a global journal that offers expert knowledge in process control and food safety and covers topics such as food process control, food safety for human consumption, mycotoxins, microbial food safety, HACCP, food safety quality assurance or risk assessment. Most of the top prolific publishers (Table 9) are part of Quartile 1 of Scopus and have been included in this database at least since 2000. Some of the top publishers on the subject of HACCP and modern quality management systems in the food industry are books that include chapters on this topic indexed in Scopus. These are the publications for which there is no available information regarding Quartile, cite score, Cite Score Tracker, SJR or SNIP.

**Table 9:** Top prolific publishers.

Sources	NP on topic	Q	Cite score 2021	Cite Score Tracker 2022	SJR	SNIP 2021	Scopus coverage years
Food Control	34	Q1	9.3	9.9	1.083	1.708	1990 to 2023
Acta Horticulturae	11	Q1	0.5	0.5	0.163	0.226	1976, 1988, from 1996 to Present
International Journal of Food Microbiology	8	Q1	9.5	9.7	0.997	1.579	from 1984 to 2023
British Food Journal	6	Q1	4.3	5.0	0.609	0.984	from 1899 to Present
IOP Conference Series: Earth and Environmental Science	6	Q4	0.6	0.8	0.202	0.409	from 2010 to Present
Contributions to Management Science	4	Q4	0.7	0.7	0.130	0.000	from 2005 to 2023
Food Reviews International	4	Q1	12.9	14.0	0.962	1.817	from 1985 to Present
Food Safety Management: a Practical Guide for the Food Industry	4	N/A	N/A	N/A	N/A	N/A	Book published in 2004
International Journal of Environmental Health Research	4	Q1	5.0	5.5	0.571	1.038	from 1991 to Present
Swainson's Handbook of Technical and Quality Management for the Food Manufacturing Sector	4	N/A	N/A	N/A	N/A	N/A	Book published in 2018

NP – number of publications; Q – quartile; N/A – no available information; Cite Score - evaluates the journal impact by calculating for the current year the total number of citations a journal has received during the last four years divided by the total amount of papers published in that journal during the four years. CiteScore Tracker – is a current evaluation of a journal's performance over the course of the year updated every month. SJR - the SCImago Journal Rank takes into consideration the number of citations a journal receives and the reputation of the journals from which those citations originate, estimating the prominence of scientific publications. SNIP - the number of citations received to publications issued in the previous three years divided by the total number of works published in the previous three years.

**Fig. 9:**
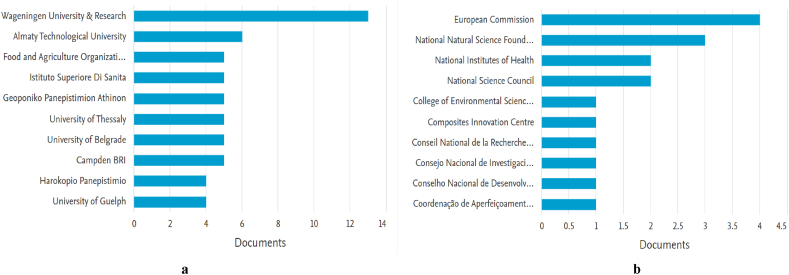
a Documents by affiliation – Scopus. b Documents by funding organization – Scopus.

To analyze the distribution characteristics of the major research affiliations (Fig. 9a) and founding organizations (Fig. 9b) data from Scopus database was evaluated and illustrated in the figures below. Most articles were published by authors affiliated with Wageningen University & Research (The Netherlands), namely 13 documents, followed by Almaty Technological University (Kazakhstan) with six publications. The other universities have a fairly small number of affiliated authors who have published scientific production on HACCP and modern quality management systems in the food industry. However, they mostly belong to the countries where the most prolific corresponding authors come from, namely Italy or Greece.

Regarding funding sponsors, the main institution is the European Commission with four documents, followed by the National Natural Science Foundation of China with three affiliated documents, National Institutes of Health (USA) and National Science Council (USA), both with two documents each. It should be noted that most publications do not have their affiliation included in the published documents, not being sponsored by foundations, so this information is not found in all articles. There are 328 papers that do not have the funding sponsor included.

**Fig. 10:**
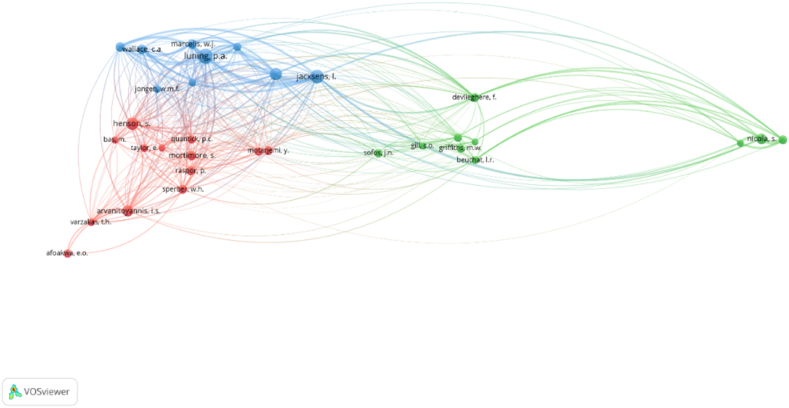
Science mapping - intellectual structure: Co-citation Network Graph.

Bibliographic coupling usually develops while publications cite the same work. This can show the relative merits of a certain publication to a corpus of related works and the approach can be utilized with publications, journals, scholars, institutions, and nations. By examining the bibliographic coupling of authors, it is feasible to determine which publications and writers are connected by many citations. For this stage of our research, we developed a co-citation analysis using the VOSviewer software with the aim of investigating the most-cited scholars. The most popular method of evaluating the impact of authors, journals, and publications is through the examination of article citations since it identifies the relevant authors in the field of study [67,78]. Fig. 10 shows the bibliographic coupling between scholars and it was generated imposing the following restrictions in the VOSviewer software: the chosen unit of analysis is cited authors, while the counting method is full counting. 25 is the minimum number of citations of an author. Based on these restrictions, out of 17,967 cited authors, 32 met the threshold. The results generated three clusters, 359 links with a total link strength of 16,039. The three clusters of cited authors are illustrated in Fig. 10 with green, blue and red colors.

**Fig. 11:**
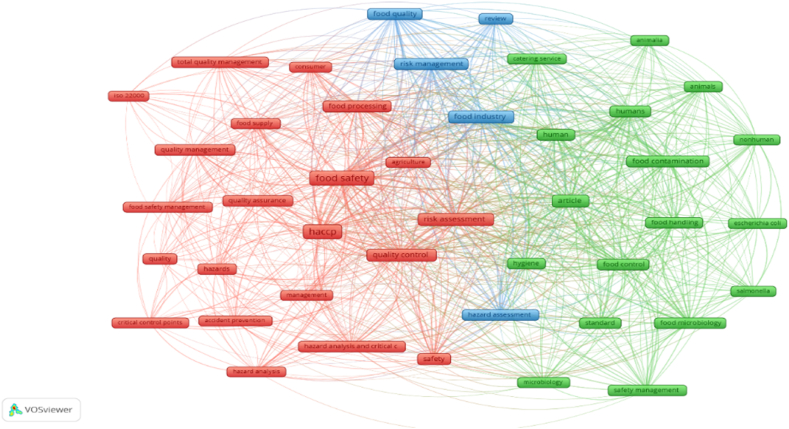
Science mapping - conceptual structure: Co-word analysis.

Research question five aims to clarify how do the keywords referring to HACCP and modern quality management systems in the food industry cluster together. Keyword analysis illustrates relevant research areas and identifies interlinks in various topics of research. Keyword analysis can rapidly and reliably capture the issue of HACCP and modern quality management systems in the food industry since keywords serve as a high-level summary of the articles’ subject. The keyword analysis was evaluated using VOSviwer and it is illustrated in Fig. 11. VOSviwer provides three types of keywords analyses, namely „All keywords”, Author keywords” and „Index keywords”. For this research, we used „All keywords” option, „Co-occurrence” as type of analysis and „Full counting” method. The minimum number of occurrences of a keyword is set at 15 and this generated 43 results that met the threshold, out of 2259 keywords. These formed three clusters: red, green and blue, 751 links and 4844 total link strength.

Table 10 includes the evaluation of the three clusters above mentioned. The best-represented cluster is the red one, counting 27 items related to HACCP, quality management and safety. Some of the most used words included in this cluster are: “food safety”, “HACCP”, “quality control” or “risk assessment”. Cluster number two, represented in green, includes 17 items and the ones with the most occurrences are: “article”, “food contamination”, “human” or “food control”. The third cluster is represented in blue and counts five items, among the most used being: “food industry”, “food quality”, “hazard assessment” or “risk management”.

**Table 10:** Author keyword clusters.

Cluster	Number of Items	Main Keywords	Occurrences	Total Link Strength
Cluster 1 (red)	27 items	Food safety	152	733
		Food processing	37	250
		HACCP	137	498
		Quality control	68	431
		Quality management	26	108
		Risk assessment	60	461
		Safety	39	249
		Hazard analysis and critical control points	27	186
Cluster 2 (green)	17 items	Article	64	519
		Food contamination	45	423
		Food control	33	325
		Food microbiology	29	275
		Human	43	386
		Humans	36	371
		Safety management	19	176
Cluster 3 (blue)	5 items	Food industry	62	463
		Food quality	44	344
		Hazard assessment	33	263
		Risk management	37	287

**Fig. 12:**
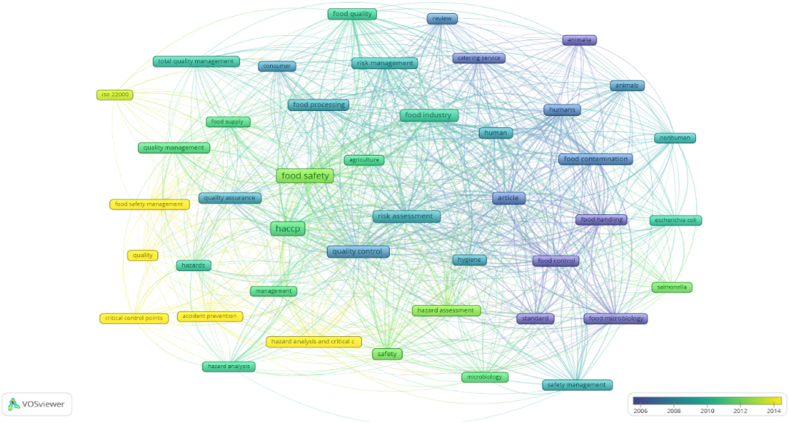
Evolution of science mapping - conceptual structure: Co-word analysis.

Fig. 12 shows the development of the co-word analysis of the conceptual structure on science mapping based on HACCP and modern quality management systems in the food industry. It should be noted that terms included in Cluster 1 are present in more recent research in comparison to many of the words in cluster 2, which are found more in scientific production from before 2010. It is noteworthy the inclination of scholars in recent years for aspects related to quality, food safety management, critical control points or accident prevention.

The sixth research question aims to provide information regarding the main future research directions regarding the analyzed topic. In this sense, the thematic evolution option from Bibliometrix software was used. Theme mapping is a technique that combines the benefits of conventional indexing and artificial intelligence with the intention of exploring and justifying research themes. The issues brought on by a big volume of disorganized information can be resolved through the theme map. The workload and significance of the topic are represented by the horizontal and vertical axes, which show centrality and density, respectively. The position of a theme's quadrant on the subject map can be used to determine where it is in its present development [88].

**Fig. 13:**
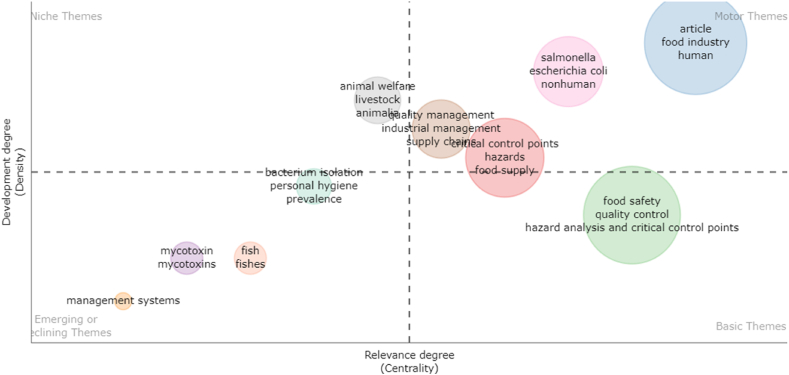
Thematic evolution.

The thematic map is divided into four quadrants: niche themes, emerging or declining topics, basic themes, and motor themes, depending on the importance and degree of growth of the topic (Fig. 13). Niche themes, while well-developed, are less significant to the area of HACCP and modern quality management systems in the food industry. These include: animal welfare, livestock and animalia, all three having the tendency to move to the motor themes quadrant. Motor themes are significant and soundly trending, reflecting the main interest of the researchers for the topics in the analyzed domain. Here, there are included several areas, such as food industry, main food diseases, quality control, hazards or food supply. Basic themes in the are significant for HACCP and modern quality management systems in the food industry and have space for development. Here, there are included food safety, quality control and HACCP. Emerging or declining topics in the last quadrant are minor, with little workload and importance. These include several less important topics such as fish, mycotoxins or personal hygiene.

To analyze the main future research directions regarding the analyzed topic, Word Dynamics from the Bibliometrics program was used using the database downloaded from Scopus. This organizes the words most often found in the articles in the database according to the year of use. According to data processing, the most relevant words in the trend are food safety, having the most appearances in scientific production in recent years, followed by quality control and risk assessment (Table 11). From the list of the 10 most frequently found words in publications, on the last place is hazard assessment, which has 37 occurrences in the year 2022. Most of the words generated by the Bibliometrix software are found as high occurrences in Fig. 12.

**Table 11:** Word dynamics (Source: data processed by authors in Bibliometrix).

Year	food safety	quality control	risk assessment	article	food contamination	food industry	food handling	food quality	human	hazard assessment
2022	121	95	76	64	63	59	48	44	44	37
2021	119	93	75	62	63	58	48	43	43	36
2020	109	92	74	60	62	52	48	41	43	32
2019	105	88	74	60	62	52	48	40	43	30
2018	99	86	68	57	61	50	48	39	40	30
2017	95	83	68	56	61	50	46	39	39	30
2016	89	81	68	53	59	49	44	38	37	30
2015	83	75	65	52	56	47	42	34	32	27
2014	76	73	62	50	53	46	42	31	31	25
2013	62	66	58	48	46	42	40	28	28	22

## 5 Limitation and future research directions

The results of this study, including the research framework, may be useful to academics and to a broad range of organizations in the food industry looking to implement a change that produces insightful analysis.

By using more databases and visualization techniques, the resulting outcomes may be expanded. The usage of additional beneficial visualization tools like Bibexcel, Gephi, or Pajek is an option. Therefore, useful data may be provided to offer specific answers to study topics. In the near future, a more thorough analysis might be conducted by involving other databases such as Web of Science in order to gather more data insights on the search themes. The breadth of the study and the subject's expandability can both be expanded with the addition of fresh information and sources. This research can be updated using the same methodology as the corpus of papers and studies grows, adding fresh overlays and ultimately strengthening the subject.

The main future research directions regarding food quality and safety management are the following:

• Assessing the impact of food labeling and consumer education on food safety: There is a need to more accurately assess the impact of food labeling and consumer education efforts on consumer food safety practices and knowledge. Research could explore consumer education campaigns to better inform consumers about food safety practices.
• Implementation of digital technologies: The use of digital technologies, such as the Internet of Things (IoT), can provide real-time monitoring and analysis of food production processes, and can help to identify and mitigate potential quality issues. Moreover, research is needed to explore innovative technologies, such as robotic systems, that can help to improve food safety management systems and reduce the risk of foodborne illness.
• Exploration of emerging risks: As the food industry continues to evolve, emerging risks, such as those related to novel food products or changing consumer preferences, will need to be identified and addressed.
• Investigating new technologies and processes to improve food safety: There is a need to assess the effectiveness of new technologies and processes in improving food safety, such as computerized monitoring systems and robotics, in industrial food production and processing.
• Assessing the efficacy of risk communication and risk assessment strategies in practice: There is a need to better understand how risk communication and risk assessment strategies can be used in practice to ensure food safety. Research is also needed to assess the effectiveness of the different communication strategies and their impact on consumer food safety.
• Researching improved methods to store and transport food safety: Research is needed to identify the most effective methods for storing and transporting food safely, and evaluating the impact of improved storage and transport practices on food safety.
• Exploring different methods to manage food safety across international food systems: Research is needed to identify the best strategies to effectively manage and monitor food safety measures across different countries, cultures, and food systems.
• Assessing the effectiveness of food safety regulations: Research is needed to assess the effectiveness of food safety regulations, and to identify ways to improve compliance with these regulations.
• Investigating the impact of food labeling and consumer education on food safety: Research is needed to explore the impact of food labeling and consumer education efforts on consumer food safety practices and knowledge.

Overall, future research in the field of quality management and HACCP will be focused on improving the safety, quality, and sustainability of food products, while also adapting to changing consumer demands, emerging risks, and regulatory requirements.

## 6 Conclusions

In order to shed light on the state of research and trends in the field and give researchers and industry representatives a foundation for further work on HACCP and modern quality management systems in the food industry and identifying the barriers and opportunities of implementing HACCP in organizations, the goal of this study was to analyze the scientific production on the HACCP and modern quality management systems in the food industry in the time frame 1978–2022 using a bibliometric approach as well as a systematic review.

The objective of this article was to carry out a global review of HACCP trends as well as to highlight the relevant topics covered in the research papers on HACCP and on the modern quality management systems within the food industry. Main findings illustrate that scientific production in the field of HACCP and modern quality management systems in the food industry intensified after the 1990s, with the USA being most prolific affiliation terms of scientific production until 2012. Recent studies from Italy, the United Kingdom, China and Greece intensified and currently, the most prolific country in terms of publications is Italy. The most active nations in terms of global cooperation are the UK, The USA and The Netherlands. Main motor themes that reflect the main interest of the researchers refer to food diseases, quality control, hazards or food supply. Emerging or declining topics include several less important topics such as fish, mycotoxins or personal hygiene. Future research should be focused on improving the safety, quality, and sustainability of food products, while organizations adapt to evolving consumer requirement, emerging risks, and regulatory requirements.

The bibliometric analysis is of interest to researchers as well as to companies involved in the food industry. The scientists can find in this work discussion topics treated by other professionals, recommendations for addressing other topics of interest in this field, but also proposals for a multidisciplinary approach to science in order to determine new theories and methodologies that are relevant to the food safety.

## Funding

This research received no external funding.

## Author contribution statement

Elena Radu, Adriana Dima, Ecaterina Milica Dobrota, Ana-Maria Badea, Dag Øivind Madsen, Cosmin Dobrin, Silvius Stanciu - Conceived and designed the analysis; Analyzed and interpreted the data; Contributed analysis tools or data; Wrote the paper.

## Data availability statement

Data will be made available on request.

## Additional information

No additional information is available for this paper.

## Declaration of competing interest

The authors declare that they have no known competing financial interests or personal relationships that could have appeared to influence the work reported in this paper.

## Appendix A

Research protocol combining bibliometric analysis and systematic review.
